# Effect of dissolved humic acids and coated humic acids on tetracycline adsorption by K_2_CO_3_-activated magnetic biochar

**DOI:** 10.1038/s41598-022-22830-9

**Published:** 2022-11-08

**Authors:** Meifang Li, Ping Wang, Chenxi Huang, Yunguo Liu, Shaobo Liu, Ke Zhang, Jingxiao Cao, Xiaofei Tan, Shaoheng Liu

**Affiliations:** 1 College of Environmental Science and Engineering, Central South University of Forestry and Technology, Tianxin District, Shaoshan South Road, Changsha, 410004 People’s Republic of China; 2 Faculty of Life Science and Technology, Central South University of Forestry and Technology, Changsha, 410004 People’s Republic of China; 3 College of Environmental Science and Engineering, Hunan University, Lushan South Road, Yuelu District, Changsha, 410082 People’s Republic of China; 4 Key Laboratory of Environmental Biology and Pollution Control, Ministry of Education, Hunan University, Lushan South Road, Yuelu District, Changsha, 410082 People’s Republic of China; 5 School of Architecture and Art, Central South University, Lushan South Road, Yuelu District, Changsha, 410083 People’s Republic of China; 6 Department of Chemistry and Chemical Biology, Northeastern University, 360 Huntington Ave, Boston, MA 02115 USA; 7 College of Chemistry and Material Engineering, Hunan University of Arts and Science, Dongting Avenue, Wuling District, Changde, 415000 Hunan People’s Republic of China

**Keywords:** Nanoscale materials, Environmental impact

## Abstract

Humic acids (HAs) widely exist in water environment, and has an important impact on the adsorption of pollutants. Herein, HAs (both dissolved and coated) was employed to assess the effect on the removal of the organic contaminant tetracycline (TC) by K_2_CO_3_ modified magnetic biochar (KMBC). Results showed that low concentration of dissolved HAs promoted TC removal, likely due to a bridging effect, while higher concentration of dissolved HAs inhibited TC adsorption because of the competition of adsorption sites on KMBC. By characterization analysis, coated HAs changed the surface and pore characteristics of KMBC, which suppressed the TC removal. In a sequential adsorption experiment involving dissolved HAs and TC, the addition of HAs at the end of the experiment led to the formation of HAs-TC ligands with free TC, which improved the adsorption capacity of TC. TC adsorption by KMBC in the presence of dissolved HAs and coated HAs showed a downward trend with increasing pH from 5.0 to 10.0. The TC adsorption process was favorable and endothermic, and could be better simulated by pseudo-second-order kinetics and Freundlich isotherm model. Hydrogen bonds and *π*–*π* interactions were hypothesized to be the underlying influencing mechanisms.

## Introduction

Biochar (BC) is a porous carbon material prepared by pyrolysis of biomass, such as plant residues and animal wastes^1,2^. It is considered a promising alternative adsorbent for wastewater treatment due to high porosity, thermo-stability, low cost, and recycling potential^3^. Biochar has shown high efficiency in the adsorption of a wide range of contaminants spanning heavy metals and organic pollutants^4,5^. Despite these advantages, the effective utilization of the pristine biochar in environmental remediation can still be improved, especially in solid–liquid separation and adsorption capacity, due to the scarcity of their surface functional groups^6^.

The introduction of magnetic nanoparticles to biochar surface can yield improved solid–liquid separation properties, which, however, occurs at the cost of reduced adsorption capacity due to the occupancy of the adsorption sites by magnetic nanoparticles^7^. To circumvent this problem, surface chemical modification of biochar has been proposed, which activates biochar for specific adsorption functions^8^. To date, various types of chemical reagents have been applied to the surface activation of biochar, such as ZnCl_2_, MgCl_2_, KMnO_4_, H_2_SO_4_, H_3_PO_4_, KOH, and K_2_CO_3_^9–11^. Among these reagents, K_2_CO_3_ is not deleterious to human health and has been used as food additives. Moreover, modification of biochar with K_2_CO_3_ activation has been shown to significantly improve surface area, pore volume, and aromaticity^12^. Therefore, K_2_CO_3_ is a highly applicable biochar activation agent.

The biochar-based nanocomposites have been used for the removal of organic pollutants, for example, antibiotics^13^, dyes^14^, and pesticides^15^. As a typical class of organic contaminants, antibiotics are frequently detected in surface water, ground water, and drinking water^16^. Overuse of antibiotics increases the risk of bacterial drug resistance, resulting in the most common antibiotics no longer being able to effectively control infectious diseases. Concerns have also been raised about antibiotics and antibiotic resistance genes (ARGs), which may impact the structure and the activity of environmental microbial populations^17^. Moreover, once ARGs are successfully integrated in gene-transmission elements, they can persist in and transmit even in the absence of selection pressure^17,18^. Thus, the removal of antibiotic contaminants is of significant practical importance, and biochar and its derivatives has been validated for this purpose^4,9,19–21^.

Nevertheless, pollutants in real-world water environments are not isolated, and other substances often affect the removal of pollutants. Humic acids (HAs), as a ubiquitous dissolved organic matter (DOM), consists of numerous functional groups including carboxylic, phenolic and aromatic groups, which can be modulate the interactions between biochar and pollutants. For example, HAs may alter the physicochemical properties of biochar, change their surface reactivity, and affect its adsorption behavior to multifarious contaminants^22^. In most cases, adsorption of organic contaminants in water by biochar is strongly inhibited by the coexisting HAs through pore plugging and competitive adsorption sites^23^. In contrast, there were some reports demonstrating that the adsorption of antibiotics on unmodified biochar could be improved in the presence of HAs^24^. However, related mechanistic research of HAs effect on the adsorption of antibiotics by modified biochar is lacking. In addition, there are few studies on the effect of HAs on the aggregation or autooxidation of magnetic nanoparticles, and the related mechanism is not clear.

Herein, we introduce a novel biochar composite, K_2_CO_3_-modified magnetic biochar (KMBC), which was prepared through a simultaneous activation and magnetization process. Tetracycline (TC) was selected as model antibiotic because of its long-term use in farmland and frequent detection in drinking water. The main objectives of this investigation were: (1) to explore the effect of dissolved/coated HAs on TC removal by KMBC; (2) to study the effect of pH and addition sequences of HAs on TC removal by KMBC; (3) to clarify the interaction and potential mechanisms of KMBC for TC uptake in the presence of HAs. This work is expected to provide new insights into how HAs interacts with modified biochar and organic contaminants in wastewater.

## Materials and methods

### Materials

Rice straw in this study was collected from the waste biomass of the farm of Yiyang, Hunan province, China. Hydrochloride salt of tetracycline (TC) was obtained from Hefei Bomei Biotechnology Co., Ltd., China. Humic acids (HAs) were purchased from Shanghai Chemical Corp. (Shanghai, China), which was purified before use, and the purification method referred to Wang et al.^25^ (described in the Supplementary Materials). All other chemicals used in this work were analytical grade and solutions were prepared using Milli-Q water (18.2 MΩ/cm) (Millipore, Billerica, MA) in this work.

### Preparation of RBC, KMBC, HAs/KMBC

The collected rice straw was rinsed with Milli-Q water several times and air-dried, then ground into powder and sieved through a 100-mesh sieve. The unmodified rice straw biochar (RBC) was pyrolyzed in a tubular furnace with a 5 °C/min heating rates to 600 °C, which was maintained for 2 h in a N_2_ atmosphere, before being cooled to room temperature. To prepare K_2_CO_3_ activated magnetic biochar (KMBC), desired amounts of K_2_CO_3_ was added into the mixed solution of 10 g rice straw and 2 g FeCl∙6H_2_O (50 mL) and stirred at 25 °C and 150 rpm for 24 h. The mixture was dried at 80 °C and then pyrolyzed at 600 °C for 2 h in a N_2_ atmosphere. The samples were washed repeatedly with deionized water, followed by drying at 60 °C.

KMBC was pre-coated with purified HAs at a mass ratio of 1% and 10% (HAs: KMBC), named as 1% HAs/KMBC and 10% HAs/KMBC. HAs/KMBC was synthesized with the following steps. Different amounts of purified HAs were dissolved in 25 mL Milli-Q water, followed by adjustment of pH to 7 with NaOH or HCl. Then, 0.5 g KMBC was added into the solution. The samples were shaken at 25 °C with 170 r/min for 24 h and kept quiescent for 48 h, washed with Milli-Q water for three times, and freeze-dried in vacuum^24^.

### Batch experiments

The effect of the ratio of K_2_CO_3_ to rice straw (0.5:1, 1:1, 2:1, and 4:1) on TC adsorption was explored in 25 mL 50 mg/L TC solution. For subsequent experiments, a fixed ratio (K_2_CO_3_: rice straw) of 4:1 (w/w) was adopted. In a typical adsorption experiment, a 50 mL conical flask was charged with 25 mL 50 mg/L TC solution as adsorbate and 0.005 g RBC, KMBC, HAs/KMBC as adsorbent. The sorption kinetics was studied at 150 rpm at 25 °C from 0 to 26 h. In the adsorption isotherm and thermodynamics experiments, the initial concentrations of TC were 50–300 mg/L under three different temperatures (25 °C, 35 °C, and 45 °C). The pH effect in the reaction system was set as 5.0–10.0 with adjusted with 0.1 mol/L HCl and NaOH solution. All of the experimental data were the average of twice or three duplicate experiments.

Influence of dissolved HAs on 50 mg/L TC uptake was studied at different HAs concentrations in the range of 0.5–20 mg/L. The effects of addition sequences of dissolved HAs and KMBC on the sorption of TC were investigated in the pH range of 5.0–10.0. The addition sequences were as follows: (1) KMBC and 5 mg/L HAs at pH 5.0–10.0 were pre-equilibrated for 26 h before adding TC (denoted as (KMBC-HAs)-TC); (2) KMBC and 50 mg/L TC at pH 5.0–10.0 were pre-equilibrated for 26 h before adding dissolved HAs (denoted as (KMBC-TC)-HAs); (3) The mixture of 50 mg/L TC and 5 mg/L HAs at pH 5.0–10.0 were pre-equilibrated for 26 h before adding KMBC (denoted as (TC-HAs)- KMBC).

### Detection of TC and HAs

The concentrations of TC in the supernatants were analyzed by HPLC (Agilent 1100, USA) on a C18 column (4.6 × 150 mm) with UV–visible detection at a wavelength of 357 nm. The mobile phase consisted of 0.01 M oxalic acid: chromatographic acetonitrile (v/v, 4:1) at a flow rate of 1.0 mL/min. The injection volume was 20 μL, and the column temperature was 30 °C^26^. The residual HAs concentration in the supernatant was determined by a UV–visible spectrometer (UV-2550, SHIMADZU, Japan) at 254 nm^24^.

### Characterization of adsorbents

The surface structure and elemental composition was analyzed by scanning electron microscopy (SEM) (Quanta 400 FEG, USA) and energy disperse X-ray spectroscopy (EDS) (Genesis, USA). Magnetic properties were measured on a vibrating sample magnetometer (VSM) (Lake Shore 7410, USA). The surface elemental composition and elemental species were characterized by an X-ray photoelectron spectroscopy (XPS) using an ESCALAB 250Xi spectrometer (Thermo Fisher, USA). The surface functional groups were recorded on Fourier transform infrared spectrum (FTIR) using an IRTracer-100 spectrometer (Shimadzu, Japan). The Brunauer–Emmett–Teller (BET) surface area and pore structure analysis of the samples were obtained by a Quadrasorb EVO instrument (Quantachrome, USA) basing on N_2_ adsorption methods. Zeta potentials of KMBC, and HAs coated KMBC were determined using a zeta potential analyzer (Nano-ZS90 Zetasizer, Malvern Instruments, UK).

### Data analysis

#### Model of Data analysis

The calculation formula of TC adsorption capacity (*q*_e_) is given in the following equation:

1 $$ q_{e} = \frac{{\left( {C_{{\text{o}}} - C_{{\text{e}}} } \right)V}}{m} $$

where *C*_o_ and *C*_e_ (mg/L) are the initial and equilibrium TC concentrations, respectively; *V* (L) is the initial volume of the TC solutions, and *m* (g) is the mass of the adsorbent used.

The equations of pseudo-first-order, pseudo-second-order and intraparticle diffusion models were as follows:

2 $$ q_{t} = q_{e} \left( {1{ - }\exp \left( {{ - }k_{1} t} \right)} \right) $$

3 $$ q_{t} = \frac{{q_{e}^{2} k_{2} t}}{{1 + q_{e} k_{2} t}} $$

4 $$ q_{t} = k_{p} t^{0.5} + C $$

where *q*_e_ and *q*_t_ are the adsorption capacity of TC adsorption on the adsorbent (mg/g) at equilibrium and at different time, respectively. *k*_1_ (1/min), *k*_2_ (g/mg min), and *k*_p_ (mg/g·min^0.5^) are the rate constants of the pseudo-first-order, pseudo-second-order, and intraparticle diffusion rate constants, respectively. *C* is the intercept of linear fitting of *q*_t_ versus *t*^0.^^5^.

The Langmuir and Freundlich models could be expressed as:

5 $$ q_{{\text{e}}} = \frac{{K_{{\text{L}}} q_{{\text{m}}} C_{{\text{e}}} }}{{1 + K_{{\text{L}}} C_{{\text{e}}} }} $$

6 $$ q_{e} = K_{f} C_{e}^{N} $$

where *C*_e_ is the equilibrium concentration of TC (mg/L); *q*_e_ is the equilibrium amount of TC (mg/g) and *q*_m_ is the maximum adsorption capacity corresponding to Langmuir model (mg/g); *K*_L_ (L/mg) and *K*_f_ [(mg/g)/(mg/L)^N^] are the constant of Langmuir and Freundlich models, respectively; N is the Freundlich constants that represented adsorption strength.

The Gibbs free energy (Δ*G*^o^) for TC uptake on adsorbents was calculated by the following formula:

7 $$ \Delta G^{{\text{o}}} = - RT\ln K_{0} $$

8 $$ \Delta G^{{\text{o}}} = \Delta H^{{\text{o}}} - T\Delta S^{{\text{o}}} $$

where *R* (8.314 J/mol K) is universal gas constant and *T* (K) is the solution temperature in Kelvin. *K*_0_ is the thermodynamic equilibrium constant, calculated by plotting ln *K*_b_ (*K*_b_ = *q*_e_/*C*_e_) versus *C*_e_ and extrapolating *C*_e_ to zero. Δ*G*^o^ can also be expressed in terms of enthalpy (Δ*H*^o^) and entropy (Δ*S*^o^) change in the adsorption process as a function of temperature. Δ*S*^o^ and Δ*H*^o^ values can be derived from the slope and intercept of Δ*G*^o^ versus *T*, respectively.

#### Analysis of variance (ANOVA)

Statistical analysis was performed with a one-way variance analysis (ANOVA) followed by the post hoc Tukey test. Duncan's statistics of F-test was used to identify the differences between experimental treatment using IBM SPSS Statistics 26.

## Results and discussion

### Characterization of samples

#### SEM and EDS analysis

**Figure 1:**
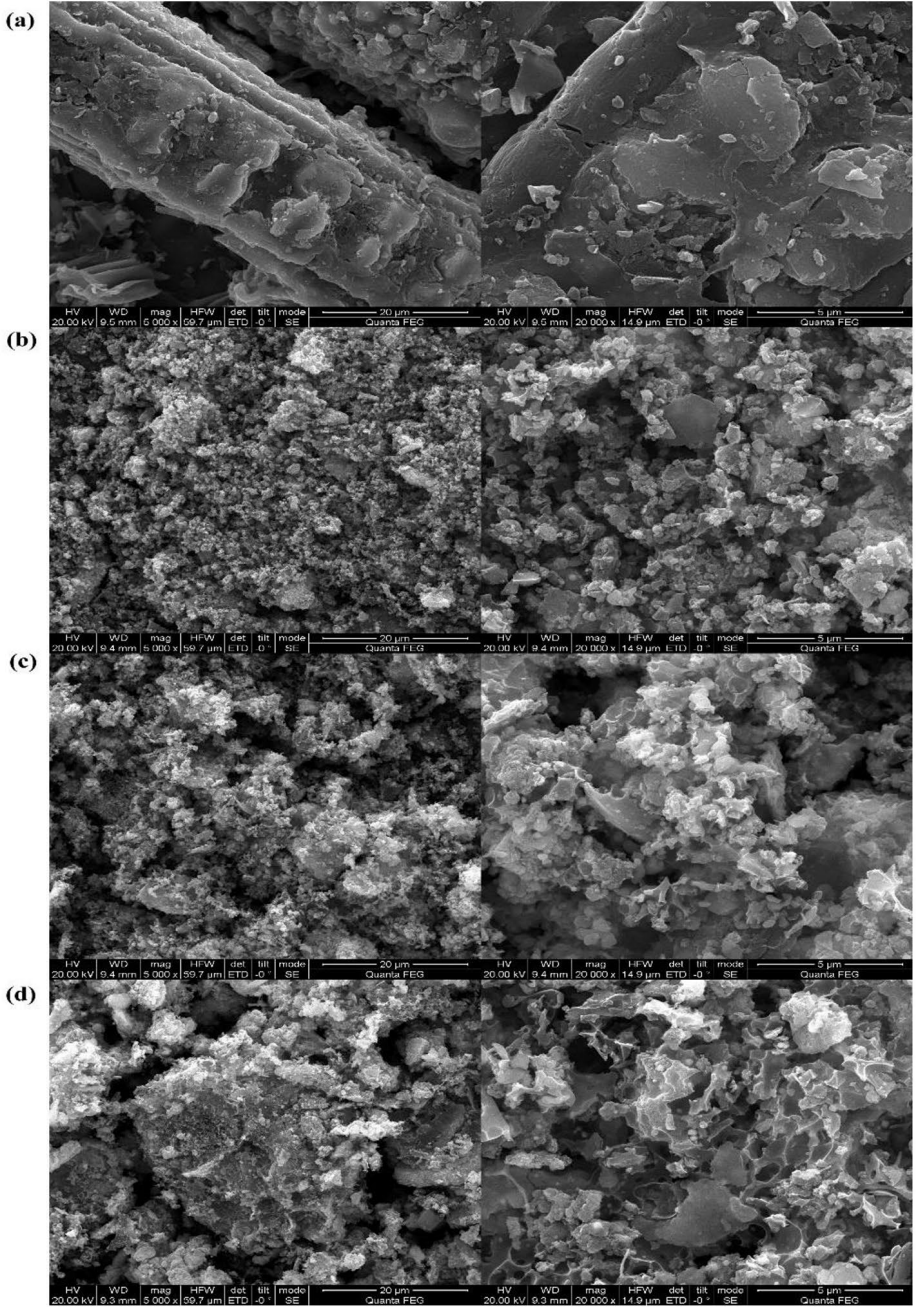
SEM images of (**a**) RBC, (**b**) KMBC, (**c**) 1% HAs/KMBC and (**d**) 10% HAs/KMBC.

Surface morphology and main elemental composition of biochar before and after modification were studied by SEM and EDS. As shown in Fig. 1, raw biochar exhibits a regular tubular structure (Fig. 1a). After activation by K_2_CO_3_, more sub-micrometer features were presented on the KMBC surface (Fig. 1b). The large number of adherents might be related to the presence of magnetic nanoparticles on the RBC surface. HAs coating further enhanced the pore structure of KMBC (Fig. 1c and d). The main elemental composition of four materials is listed in Table 1. Compared to RBC, the increased O contents of KMBC caused the surface polarity indexes [(N + O)/C] increased from 67.44 to 240.88, indicating the emergence of some oxygen-containing groups by activation. The surface polarity indexes [(N + O)/C] exhibited a reduction of 1% HAs/KMBC comparing to KMBC, which might be due to the consumption of some oxygen-containing groups due to the combination of a small amount of HAs and KMBC. However, for 10% HAs/KMBC, higher surface polarity indexes [(N + O)/C] and O content were observed, reflecting higher sorption to HAs and hydrophilicity^23^, and the increased amount of oxygen-containing groups was much greater than the combined consumption.

**Table 1:** The physicochemical properties of RBC, KMBC and different proportions of HAs/KMBC.

Sample	Surface atomic content (%)					*S*_BET_ (m^2^/g)	*V*_tot_ (cm^3^/g)	*D*_p_ (nm)
	C	N	O	Fe	(O + N)/C			
RBC	53.22	08.74	27.15	00.40	67.44	89.40	0.098	4.39
KMBC	15.07	01.90	34.40	19.30	240.88	426.15	0.402	3.77
1% HAs/KMBC	19.28	02.61	36.30	16.11	201.82	364.98	0.308	3.38
10% HAs/KMBC	15.90	02.10	44.89	12.70	295.53	503.64	0.497	3.95

*V*_tot_ is total pore volume; *D*_p_ is average pore diameter.

#### Magnetism

Good solid–liquid separation performance of adsorbent is of great significance to the real-world wastewater treatment. Thus, the magnetic hysteresis loops of KMBC, 1% HAs/KMBC and 10% HAs/KMBC were characterized (Fig. S1) to illustrate the magnetic properties of these materials. The saturation magnetization value of KMBC is 0.027 emu/g, suggesting that KMBC was sufficient to achieve solid–liquid separation with an external magnetic field. After 1% and 10% HAs coating, the magnetic behavior of KMBC was enhanced with saturation magnetization values of 0.08 emu/g and 0.06 emu/g, respectively. HAs possessed strong affinity to magnetic nanoparticles and effectively coats particle surface via the surface complexation ligand exchange reactions, which could suppress the aggregation or autooxidation of Fe_3_O_4_^27^, resulting in the rise of saturation magnetization values after HAs coating. The Fe2p peak appeared in the XPS full spectra of KMBC and 10% HAs/KMBC (Fig. S2), further confirming the successful loading of magnetic particles on KMBC and HAs/KMBC surface.

#### FTIR analysis

The FTIR analysis are shown in Fig. S4. As observed from FTIR spectra, a number of strong peaks (e.g., 3356 cm^−1^ for O–H, 1601 cm^−1^ for C = O and 1029 cm^−1^ for C–O) were identified in the spectrum of HAs, indicating it was rich in oxygen-containing groups^19,28^. These oxygen-containing groups also appeared on the RBC and KMBC. Compared with RBC, the extra peak at about 535 cm^−1^ corresponded to the Fe–O stretching vibrations in KMBC spectrum, which further confirmed that the magnetic nanoparticles were coated on the RBC surface^29^. However, after HAs loading, the oxygen-containing groups on KMBC decreased significantly, which may be the reason that KMBC might consume a lot of hydroxyl and carboxyl groups by interacting with the polar functional groups of HAs through hydrogen bonds.

### *Effect of the ratio of K*_*2*_*CO*_*3*_* on adsorbent*

**Figure 2:**
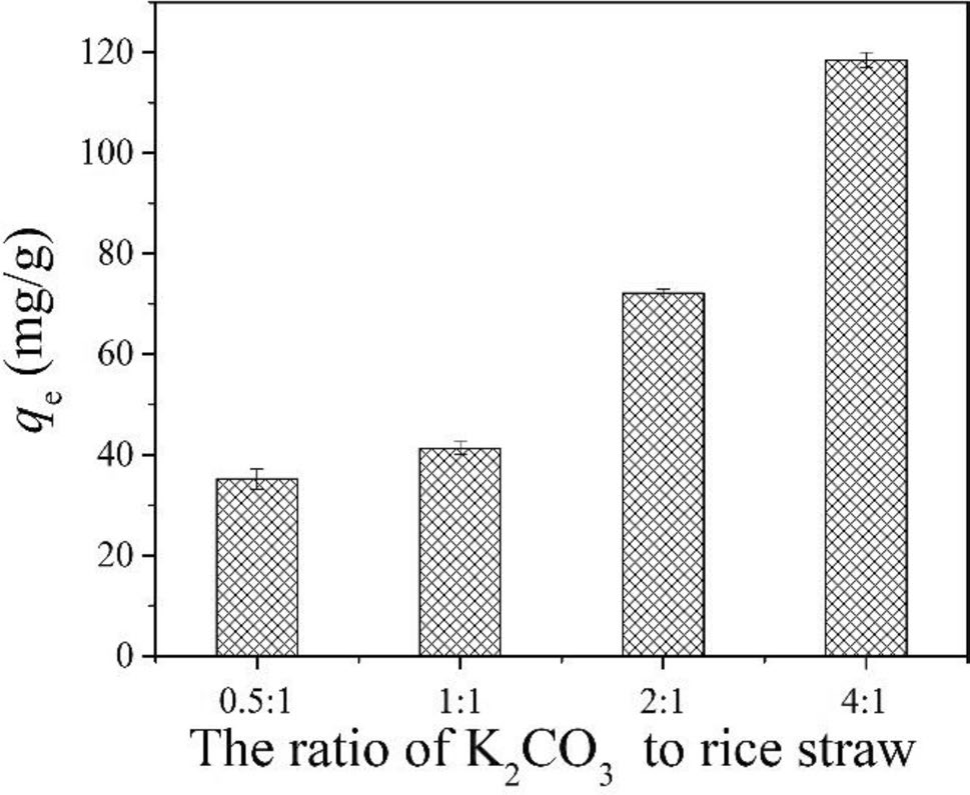
Effect of the ratio of K_2_CO_3_ to rice straw on TC removal by KMBC. *C*_0(TC)_ = 50 mg/L, *m*/*V* = 0.1 g/L, *T* = 25 °C, *t* = 26 h, pH = 7.0.

Figure 2 shows TC removal by KMBC at different K_2_CO_3_ feeding ratios at pH 7. It was found that the removal of TC increased from 35.2 to 118.4 mg/g with increasing K_2_CO_3_. The increased adsorption capacity might be related to the change of pore structure and surface properties of the modified biochar. According to the BET characterization (Fig. S3), compared to RBC, the formation of more micropores and mesopores on KMBC was observed when the ratio of K_2_CO_3_ to rice straw was 4:1. In addition, the total pore volume and specific surface area of KMBC was 4.1 and 4.8 times that of RBC (Table 1). Therefore, K_2_CO_3_ was beneficial to the enhancement of the TC adsorption performance of the materials.

### Effect of contact time

**Figure 3:**
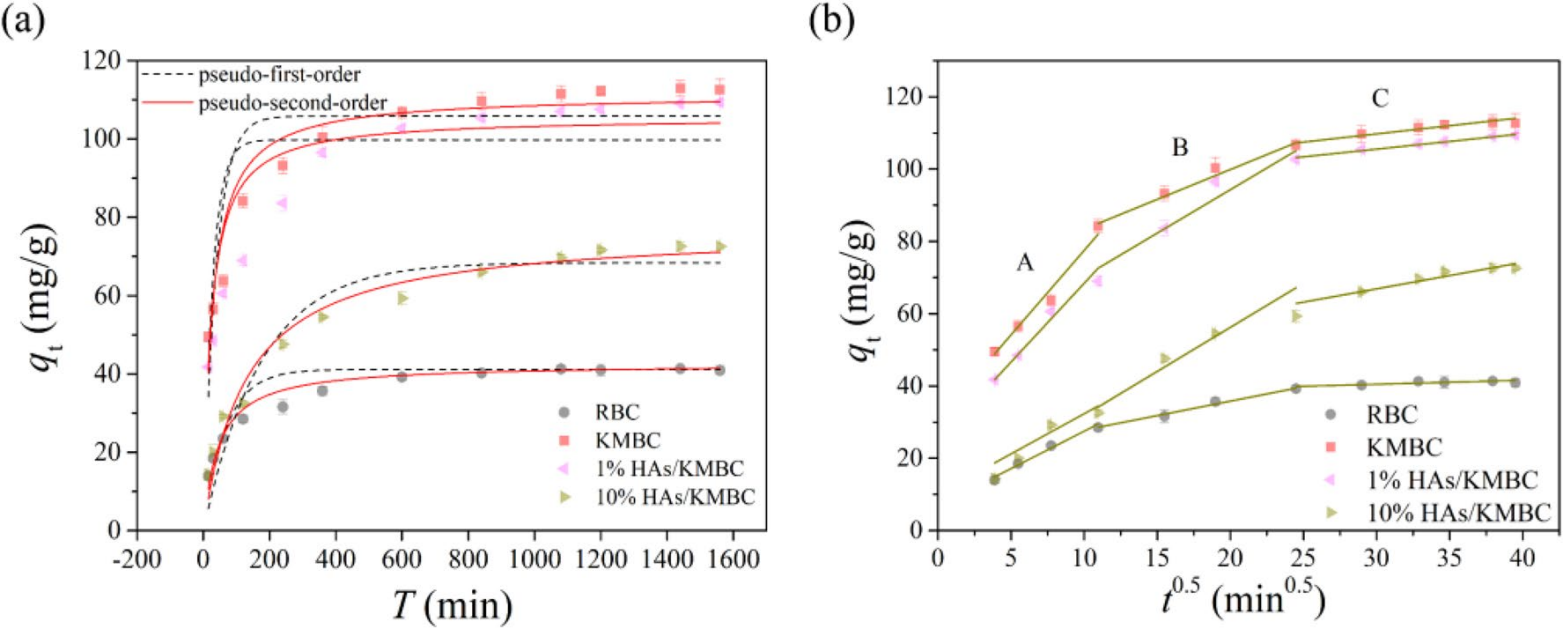
Adsorption kinetic curves of TC onto RBC, KMBC, 1% HAs/KMBC and 10% HAs/KMBC: (**a**) the solid lines and dotted lines are the pseudo-second-order model and the pseudo-first-order model simulation, respectively; (**b**) Intra-particle diffusion model simulation. *C*_0(TC)_ = 50 mg/L, *m*/*V* = 0.1 g/L, *T* = 25 °C, pH = 7.0.

Contact time is a significant parameter to evaluate the application potential of adsorbents in wastewater purification. As shown in Fig. 3, for both RBC and modified forms, the rapid adsorption of TC occurs within the first 6 h, and the adsorption rate reduced thereafter and reached equilibrium. After K_2_CO_3_ and HAs modification, longer times were needed for these systems to reach adsorption equilibrium than RBC system. These effects might be attributed to the fact that the activation process increased the specific surface area and promoted the development of pores compared to raw biochar. Besides, the competition/occupation of adsorption sites between HAs and TC might also lead to the prolongation of adsorption equilibrium. In the following experiment, 26 h was selected as the reaction time of TC adsorption to ensure that the adsorption equilibrium of all samples was established.

Pseudo-first-order and pseudo-second-order kinetic models were employed to fit the kinetic data to shed light on the adsorption mechanism. The experimental kinetic data are non-linearly fitted as presented in Fig. 3a and related parameters from the kinetic models are provided in the Table S1. Results indicated pseudo-second-order model preferably described the removal process of TC by different types of materials with higher *R*^2^. In addition, the theoretical adsorption capacity (*q*_cal_) of pseudo-second-order model was in good agreement with the experimental adsorption capacity (*q*_exp_). It showed that TC adsorption onto biochar materials were mainly controlled by chemisorption^30^.

In order to explore the diffusion mechanism of TC onto the biochar materials, the kinetic data were further analyzed by the intraparticle diffusion model based upon diffusion mass transfer. The kinetic parameters of intra-particle diffusion are displayed in Table S2. According to the values of intercept *C*, the plots showed no crossing at the origin and exhibited multi-linearity relationships within the studied contact time, demonstrating that the adsorption process was affected by more than one rate-determining step. As shown in Fig. 3b, the intraparticle diffusion plot is categorized into three regions (A, B, and C), which represented film diffusion, intraparticle diffusion, and internal surface of the adsorbent in the sorption process, respectively^31^. The intercept *C* reflected the information about the boundary layer effect^32^. Compared with KMBC, other materials exhibited decreased *C* values in all stages, demonstrating a negative contribution of TC diffusion onto HAs/KMBC; that was, HAs modification was not conducive to TC adsorption, and the effect was HAs-content-dependent.

### Adsorption isotherm and thermodynamics

**Figure 4:**
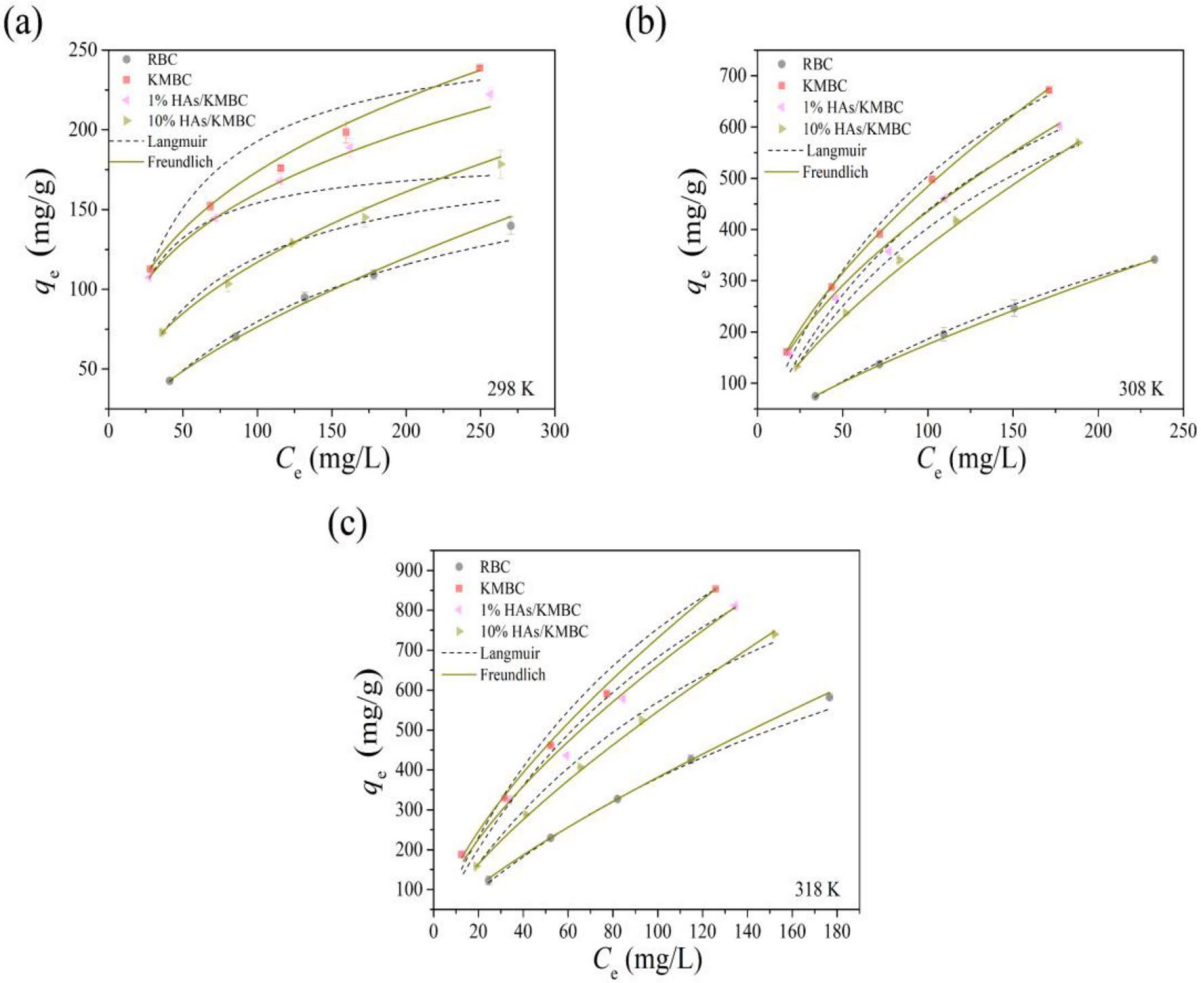
Effects of TC concentration and temperature [(**a**):298 K; (**b**):308 K; (**c**):318 K] on TC adsorption by RBC, KMBC, 1% HAs/KMBC and 10% HAs/KMBC. *m*/*V* = 0.1 g/L, *t* = 26 h, pH = 7.0.

Adsorption isotherm is used to evaluate the adsorption capacity of adsorbent and the microscopic interaction between adsorbent and adsorbate. The equilibrium adsorption data were fitted by classic isotherm models (Langmuir and Freundlich) at three temperatures (298, 308, and 318 K) and the corresponding regression parameters are listed in Table 2. As shown in Fig. 4a–c, the Freundlich model is superior to the Langmuir model for TC adsorption based on the values of *R*^2^, RMSE, and *χ*^2^. It confirmed that TC has a typical multilayer heterogeneous coverage on the interface of biochar materials^29^. Still, homogeneous adsorption cannot also be neglected in the TC removal by all four kinds of biochar materials (All *R*^2^ values in Table 2 were greater than 0.9 in the Langmuir model). According to the Langmuir model fitting, the saturated adsorption capacity (*q*_*e*_) of KMBC was higher than the *q*_*e*_ of 1% HAs/KMBC and 10% HAs/KMBC in the studied temperatures, indicating that HAs would occupy some adsorption sites and hinder TC capture.

**Table 2:** Fitting parameters of the Langmuir and Freundlich models.

Adsorbents	Temperature (K)	Langmuir model					Freundlich model				
		*q*_m_ (mg/g)	*K*_L_ (L/mg)	*R*^2^	RMSE	χ^2^	*K*_f_ [(mg/g)/(mg/L)^N^]	N	*R*^2^	RMSE	χ^2^
RBC	298	210 ± 15	(6.1 ± 0.6) × 10^–3^	0.994	1.53	2.33	3.9 ± 0.3	(64.9 ± 1.6) × 10^–2^	0.996	1.22	1.49
	308	907 ± 44	(2.6 ± 0.2) × 10^–3^	0.999	1.41	1.99	4.7 ± 0.1	(78.5 ± 0.5) × 10^–2^	0.999	0.63	0.40
	318	1365 ± 96	(3.9 ± 0.4) × 10^–3^	0.999	19.43	377.48	10.5 ± 0.3	(78.0 ± 0.6) × 10^–2^	0.999	7.73	59.82
KMBC	298	266 ± 15	(26.2 ± 2.5) × 10^–3^	0.974	11.97	143.27	36.3 ± 0.6	(34.0 ± 0.5) × 10^–2^	0.998	2.97	8.82
	308	1190 ± 93	(7.4 ± 1.0) × 10^–3^	0.992	6.70	44.89	27.9 ± 1.0	(62.0 ± 0.8) × 10^–2^	0.999	1.78	3.16
	318	1743 ± 207	(7.6 ± 1.8) × 10^–3^	0.996	21.26	452.01	32.4 ± 2.2	(67.7 ± 1.4) × 10^–2^	0.999	6.61	43.75
1% HAs/KMBC	298	185 ± 5	(50.6 ± 5.3) × 10^–3^	0.984	8.17	66.72	38.6 ± 0.5	(30.9 ± 0.3) × 10^–2^	0.999	1.25	1.55
	308	1115 ± 118	(6.5 ± 1.2) × 10^–3^	0.988	13.16	173.23	29.9 ± 3.0	(58.1 ± 2.1) × 10^–2^	0.997	6.91	47.73
	318	1684 ± 225	(6.8 ± 1.6) × 10^–3^	0.987	28.49	811.87	30.4 ± 3.3	(66.9 ± 2.3) × 10^–2^	0.997	14.30	204.58
10% HAs/KMBC	298	191 ± 5	(17.0 ± 1.2) × 10^–3^	0.996	1.73	2.98	13.9 ± 0.4	(46.2 ± 0.6) × 10^–2^	0.999	0.65	0.42
	308	1035 ± 31	(6.4 ± 0.2) × 10^–3^	0.999	10.07	101.41	15.0 ± 0.1	(69.4 ± 0.3) × 10^–2^	0.999	4.02	16.12
	318	1470 ± 129	(6.3 ± 0.6) × 10^–3^	0.993	14.14	199.89	17.5 ± 0.3	(74.8 ± 0.6) × 10^–2^	0.999	5.23	27.40

Reaction temperature is a crucial factor for inherent energy change. The effect of temperature on the TC adsorption by biochar materials was investigated at 298, 308, and 318 K (Fig. 4a–c). Van’t Hoff equation could be used to establish a relationship between the adsorption coefficient (*K*_b_) and temperature^33^, and related parameters are presented in Table S4. The negative value of Gibbs free energy (Δ*G°*) meant that the adsorption of TC on RBC, KMBC, and HA/KMBC was a spontaneous process in standard conditions^34^. Linear fitting of Δ*G*° versus *T* are shown in Fig.S6. With the rise of temperature, the value of Δ*G*^o^ declined, which indicated that the TC removal ability by four biochar materials could be improved at a higher temperature. The calculated enthalpy for all samples (Δ*H*^o^) was positive, further implying the endothermic nature of adsorption process. The entropy (Δ*S*^o^) value referred to the increase of randomness at the solid/liquid interface in the process of TC adsorption. Thus, the mechanism for TC adsorption onto RBC, KMBC, and HAs/KMBC was endothermic and energy was needed to achieve adsorption^33^.

### pH effect in the present of dissolved HAs and coated HAs

**Figure 5:**
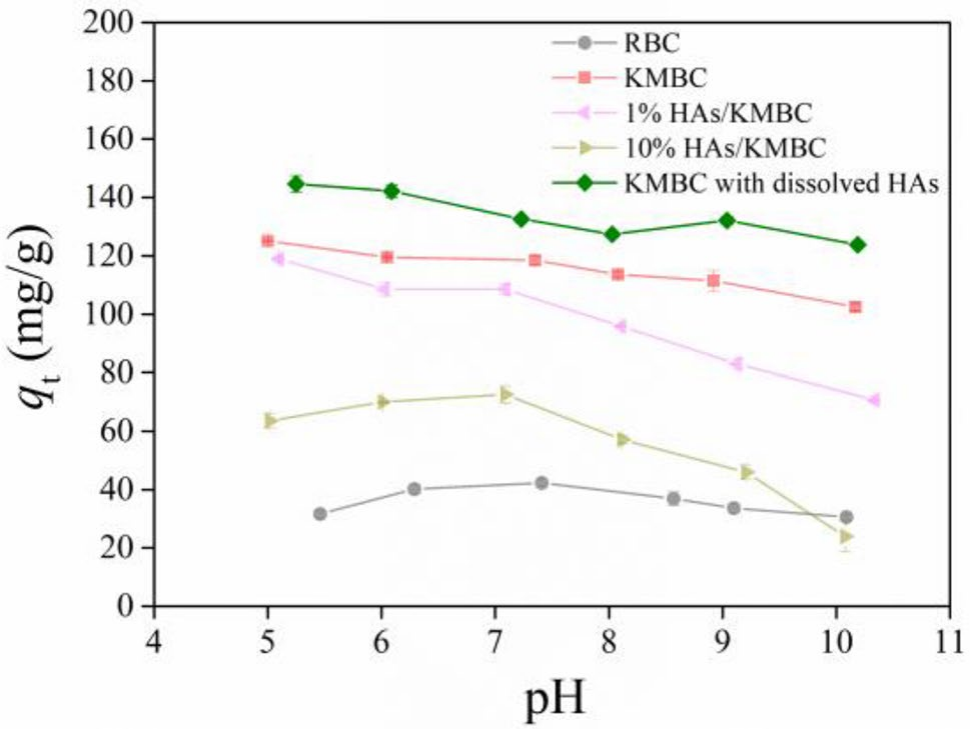
Effects of pH on TC adsorption by RBC, KMBC, dissolved HAs and coated HAs modified KMBC. *C*_0(TC)_ = 50 mg/L, *m*/*V* = 0.1 g/L, *t* = 26 h, *T* = 25 °C.

TC adsorption in the absence or presence of dissolved HAs and coated HAs as a function of pH from 5.0 to 10.0 are shown in Fig. 5. Increasing pH from 5.0 to 10.0, the TC adsorption capacity by RBC increased at first and then decreased. The optimal pH appeared at around 7.0. However, the differences were not substantial. In the same pH range, the TC adsorption by KMBC showed a downward trend, but again, the change was only 18%. The removal of TC enhanced with the addition of 5 mg/L dissolved HAs at different pH values. Thus, KMBC had the potential to be used in a broad pH range and a small amount of HAs presented in natural wastewater was conducive to TC removal in the test pH ranges. However, low or high concentrations of HAs coating had an obvious inhibition effect for TC uptake, especially under alkaline conditions. When initial pH was lower than 7.8, the surface of HAs/KMBC was negatively charged (Fig. S5b) and most TC molecules were positively charged (Fig. S5a). The electrostatic attraction might exist between the cation TC and negative HAs/KMBC surface at pH < 7.8, which was conducive to TC adsorption. However, coated HAs had inhibition effect for TC uptake at pH < 7.8, demonstrating that HAs coated on KMBC surface could occupy some the adsorption sites. The effect of occupation might be greater than the electrostatic attraction, thus limiting the adsorption of TC. When initial pH was higher than 7.8, the surface of HAs/KMBC was negatively charged (Fig. S5b). Meanwhile, TC was existed in the anion state (pH > 7.8) (Fig. S5a) in solution. Therefore, in addition to occupation, electrostatic repulsion between TC and HAs/KMBC resulted in lower TC adsorption.

### Effect of HAs concentration on TC removal by RBC and KMBC

**Figure 6:**
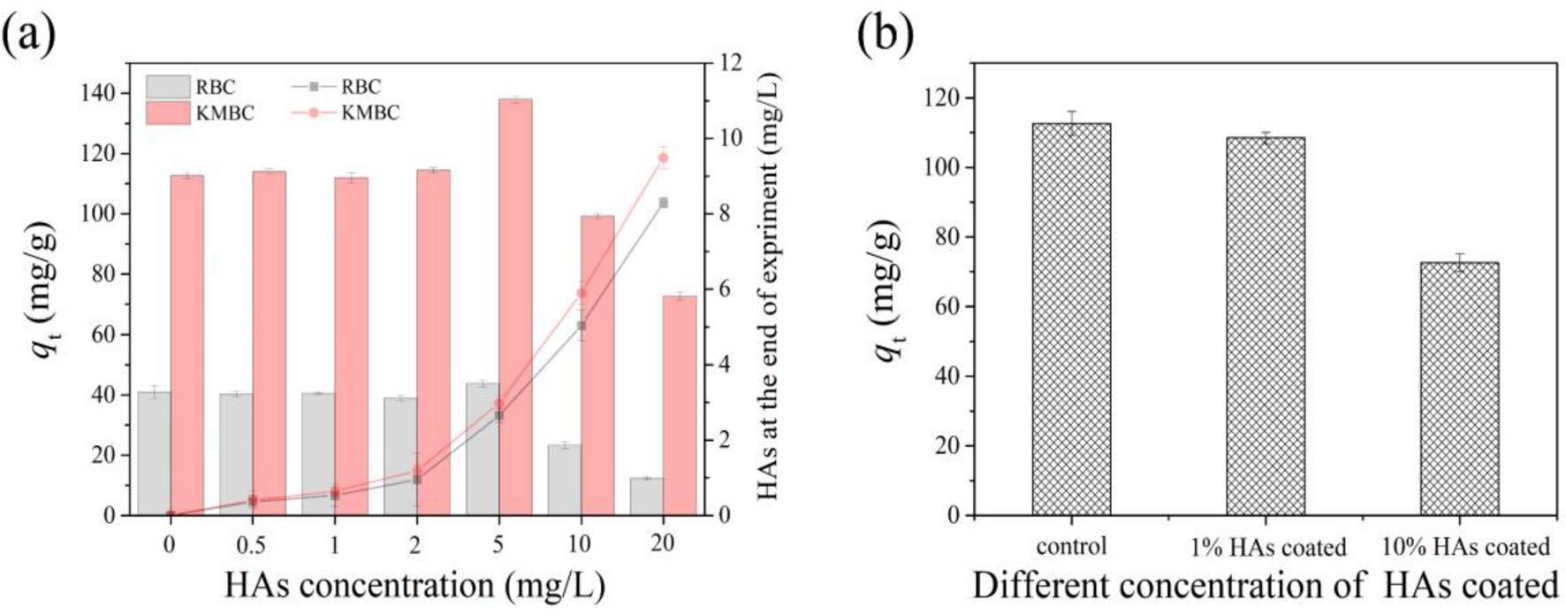
(**a**) Effects of different concentration of dissolved HAs (0–20 mg/L) on TC removal by RBC and KMBC, and the residual of dissolved HAs after reaction; (**b**) TC adsorption by different amount coated HAs modified KMBC. *C*_0(TC)_ = 50 mg/L, *m*/*V* = 0.1 g/L, *t* = 26 h, *T* = 25 °C, pH = 7.0.

The influence of different levels of dissolved HAs (0–20 mg/L) on TC removal by RBC and KMBC is presented in Fig. 6. For different HAs concentrations studied, TC sorption on RBC and KMBC was slightly improved at dissolved HAs concentration < 5 mg/L. HAs exhibited an obvious inhibitory trend when dissolved HAs concentration was > 5 mg/L. TC possessed a nitrogen aromatic heterocyclic structure, which could interact with RBC and KMBC through *π*–*π* interaction^19^. According to the BET analysis, RBC and KMBC had high porosity, thus the removal of TC could also be achieved by pore filling. Jin et al.^35^ has found that HAs could interact with TC in solution. Therefore, HAs might act as a “bridge” between the adsorbents and TC^36^ and the bridging effect might contribute to the slight initial increase in adsorption capacity at low [HAs], before being overwhelmed by the binding competition at high [HAs]. As shown in Fig. 6a, the concentration of dissolved HAs after the experiment decreased from the initial value, corroborating that that dissolved HAs was adsorbed onto the RBC and KMBC.

When HAs were coated on KMBC (Fig. 6b), TC removal was inhibited, and the inhibition was more obvious at higher HAs feed ratios. According to the BET and FTIR analysis results, the BET specific surface areas of HAs/KMBC were higher than that of KMBC, and HAs coating enhanced the polarity of KMBC and *π*–*π* interactions between HAs and KMBC (Fig. S4b), which was conducive to the adsorption of TC. However, Table 1 displays the decrease of pore diameter after low HAs coating, which probably due to pore plugging by a small amount of HAs molecules. In addition, the high coated HAs occupied the reactive sites on the KMBC, resulting in the decrease of TC adsorption. As shown in Fig. 6b, higher HAs coating led to the greater inhibition, which might be due to the more active sites being occupied.

### Effect of addition sequences of dissolved HAs and TC

**Figure 7:**
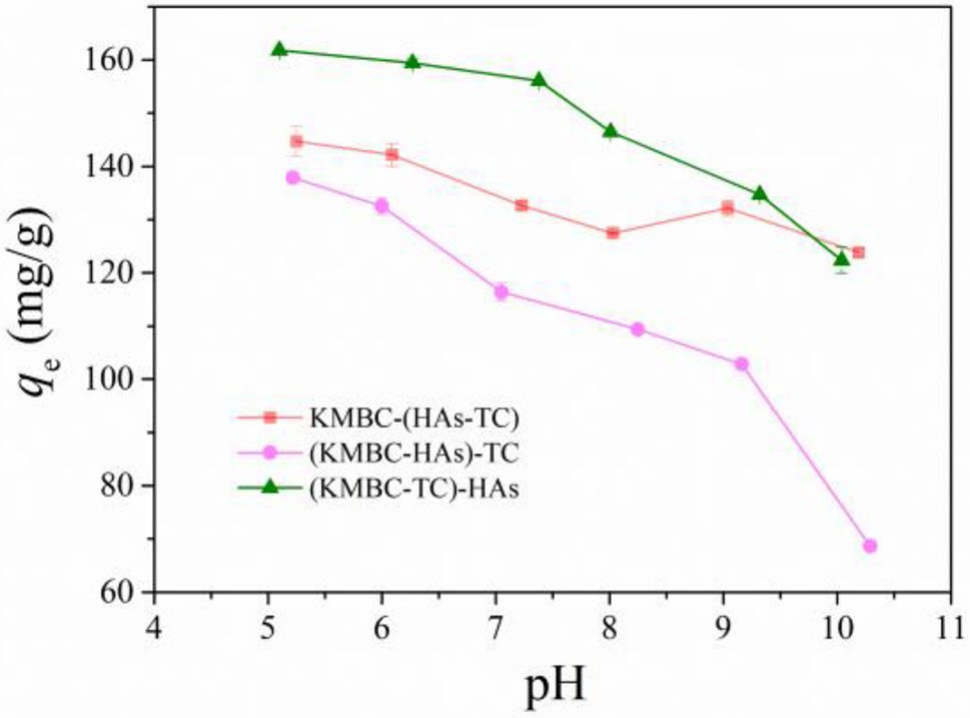
Effects of addition sequences of dissolved HAs, TC and KMBC. *C*_0(TC)_ = 50 mg/L, *C*_0(HAs)_ = 5 mg/L, *m*/*V* = 0.1 g/L, *t* = 26 h, *T* = 25 °C.

Figure 7 shows the TC sorption with the influence from HAs under three different addition sequences across the pH range 5.0–10.0. Remarkably, TC adsorption capacity was significantly influenced by the addition sequences in the pH range tested, suggesting various mechanisms existed in the complex systems of HAs, TC and KMBC.

In the (KMBC-HAs)-TC system (referring to mixing HAs with KMBC first, before adding TC), the HAs adsorbed on KMBC occupied most of the adsorption sites and pores, forming a strong hindrance which was not conducive to the subsequent capture of TC on KMBC. Hence, in this system, the removal ability of TC was lowest. In contrast, in the (KMBC-TC)-HAs ternary system, TC and KMBC were pre-equilibrated for 24 h before adding HAs, where TC had reached saturation adsorption on KMBC. When HAs was added to this mixed solution, the adsorption capacity of TC in the solution could be improved by strong combination between HAs and unremoved TC. In this case, the high adsorbed amounts of TC observed in the presence of HAs might be due to the formation of HAs-TC ligands in aqueous solution. When TC was pre-equilibrated with HAs, partial TC molecule would form complexes with HAs and then adsorb on KMBC. It was also possible that adsorption of free TC molecule might compete with free HAs for residual adsorption sites on exposed KMBC surfaces. Therefore, the TC adsorption behavior was controlled by TC-HAs complexes.

### ANOVA analysis

Statistical significance of the difference of adsorption capacities by different adsorbents under various experimental conditions were assessed. The ANOVA results (Table S3) indicated that adsorption amount of TC at most different reaction times by four adsorbents were a significant difference (*P* < 0.05). ANOVA results (Tables S5and S6) further demonstrated that initial concentration of TC and temperature had a significant effect on the adsorption of TC on the four materials (*P* < 0.05). The results in Table S7 indicate that the adsorption amount of TC by four adsorbents are significantly different in all the tested pH ranges (*P* < 0.05). It had insignificant impact on TC adsorption by four materials when the dissolved HAs concentration < 5 mg/L, while there was significant difference in adsorption amount when the dissolved HAs concentration > 5 mg/L (Table S8) As shown in Table S9, all* P* values are less than 0.05, which also suggest that the different addition sequences of dissolved HAs, TC and KMBC are statistically significant for the adsorption of TC (*P* < 0.05).

### Contribution of different mechanisms to TC removal in the presence of HAs

The adsorption mechanism could be further explained by FTIR spectra. Fig. S4b shows the FTIR spectra of HAs/KMBC before and after TC adsorption. The O–H of HAs/KMBC migrated after adsorbing TC, which might be due to the H-bond interaction, which was one of the important driving forces of adsorption. The aromatic C = C and C = O bands on HAs/KMBC surface act as π-electron-donor, which can react with π-electron-acceptor from aromatic rings of TC. The shifts of C = C and C = O bands were indicative of hydrophobic and *π*–*π* interactions between HAs and KMBC^23^.

**Figure 8:**
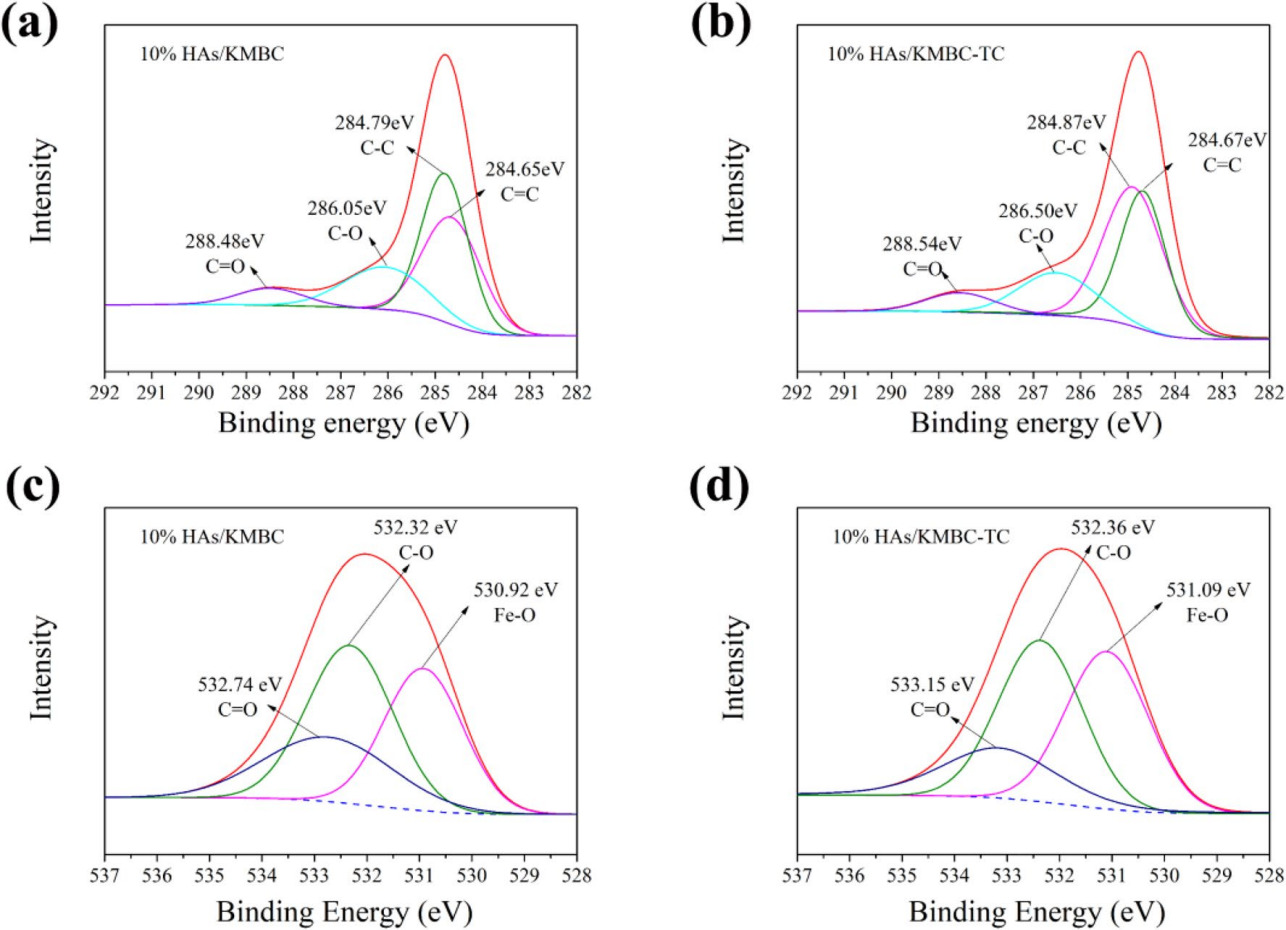
(**a**) and (**b**) are the computer deconvolution C 1 s spectra of 10% HAs/KMBC and 10% HAs/KMBC-TC, respectively; (**c**) and (**d**) are the computer deconvolution O 1 s spectra of 10% HAs/KMBC and 10% HAs/KMBC-TC, respectively.

This view was confirmed by the XPS spectra, which were used to explore the surface and internal elements of adsorbent. The computer deconvolution XPS spectra of C 1 s and O 1 s are shown in Fig. 8a–d. For 10% HAs/KMBC, the peaks of the C 1 s binding energy at about 284.65 eV, 284.79 eV, 286.05 eV and 288.48 eV were assigned to C = C, C–C, C-O and C = O, respectively^31,37^. After TC adsorption, there was a significant change of C = C peak shape, implying the aromatic C = C of HAs/KMBC might be combined with the benzene ring of TC. The O 1 s spectra of 10% HAs/KMBC was deconvoluted into three peaks at 530.92 eV, 532.32 eV and 532.74 eV, corresponding to the Fe–O, C–O, and C = O groups, respectively^29,38^. After TC uptake, the shift of C–O and C = O groups indicated that the hydroxyl and carboxyl groups of HAs/KMBC might be combined with the carbonyl and hydroxyl groups of TC, demonstrating that hydrogen bond might play a role in the adsorption process. These results were in good agreement with FTIR data.

## Conclusion

In summary, we studied a range of parameters that govern KMBC adsorption performance of TC, and investigated the potential underlying mechanisms. Sample characterization revealed that larger specific surface area and more pore structure were presented on KMBC after activation by K_2_CO_3_. BET analysis indicated more micropores and mesopores in the KMBC with increasing the amount of K_2_CO_3_, thus increasing the adsorption capacity of the materials. The VSM, FTIR and XPS analysis indicated the successful loading of magnetic particles on the RBC surface. FTIR results confirmed that HAs been loaded on KMBC via hydrogen bonds. The pseudo-second-order kinetic model and Freundlich model provided an excellent fit for the adsorption data. Thermodynamic studies demonstrated the favorability and endothermic nature of the adsorption process. Dissolved HAs provided both positive and negative influences on TC removal at different concentrations. TC removal was inhibited when HAs coating on KMBC was applied, which might be due to the more active sites occupied on KMBC surface. Sequential addition of HAs and TC revealed that both components can compete for binding with KMBC. Besides, TC-HAs complexes affected the TC adsorption behavior. According to the XPS and FI-IR analysis, the hydrogen bonds and *π*–*π* interaction were the critical driving force for this adsorption. These studies provide valuable information on biochar modification for wastewater treatment and mechanistic insight on the adsorption process, which we envision will pave the way for practical application of these materials.

## Supplementary Information

Supplementary Information.

## Data Availability

All data generated or analyzed during this study are included in this published article and its supplementary information files.
