# Supplementary material for: Effect of dissolved humic acids and coated humic acids on tetracycline adsorption by K2CO3-activated magnetic biochar
Source: Sci Rep. 2022 Nov 8;12:18966. doi: 10.1038/s41598-022-22830-9 (PMC9643364; doi:10.1038/s41598-022-22830-9)
Supplement: Supplementary file 1 — Supplementary Information. [file 41598_2022_22830_MOESM1_ESM.docx]

**Effect of dissolved humic acids and coated humic acids on tetracycline adsorption by K_2_CO_3_-activated magnetic biochar**

Meifang Li ^a,b^, Ping Wang ^a,b, *^, Chenxi Huang ^a,b^, Yunguo Liu ^c,d^, Shaobo Liu ^e^, Ke Zhang ^f^, Jingxiao Cao ^a,b^, Xiaofei Tan ^c,d^, Shaoheng Liu ^g^

^a^ College of Environmental Science and Engineering, Central South University of Forestry and Technology, Shaoshan South Road, Tianxin District, Changsha 410004, PR China

^b^ Faculty of Life Science and Technology, Central South University of Forestry and Technology, Changsha 410004, PR China

^c^ College of Environmental Science and Engineering, Hunan University, Lushan South Road, Yuelu District, Changsha 410082, PR China

^d^ Key Laboratory of Environmental Biology and Pollution Control, Ministry of Education, Hunan University, Lushan South Road, Yuelu District, Changsha 410082, PR China

^e^ School of Architecture and Art, Central South University, Lushan South Road, Yuelu District, Changsha 410083, PR China

^f^ Department of Chemistry and Chemical Biology, Northeastern University, 360 Huntington Ave, Boston, MA 02115, USA

^g^ College of Chemistry and Material Engineering, Hunan University of Arts and Science, Dongting Avenue, Wuling District, Changde 415000, Hunan, PR China

1. **HAs purification method**

First, 10 g HAs was dissolved in 400 mL 0.1 mol/L NaOH for 18 h and then centrifuged at 3500 rpm for 15 min. The supernatant was then collected. The pH of the solution was adjusted to 1-1.5 with HCl, and then centrifuged at 3500 rpm for 15 min to collect the precipitate. The collected portion was transferred to 100 mL of aqueous solution (0.5 mL HCl + 0.5 mL 48% HF + 99 mL H_2_O), then shaken for 5 h and centrifuged at 3500 rpm for 15 min. The precipitate was washed with distilled water, centrifuged and dried under vacuum at 40 °C.

**Fig. S1** Magnetization curves of KMBC, 1% HAs/KMBC and 10% HAs/KMBC.

**Fig. S2** XPS wide-scan of (a) KMBC before and after TC adsorption; (b) 10% HAs/KMBC before and after TC adsorption.

**Fig. S3** The pore size distribution of (a) RBC and (b) KMBC.

**Fig. S4** FTIR spectra of (a) RBC, KMBC, HAs-loaded samples and HAs; (b) HAs-loaded samples before and after TC adsorption.

**Fig. S5** (a) TC speciation plots in solution as a function of pH; (b) Zeta potentials of KMBC, 1% HAs/KMBC, and 10% HAs/KMBC.

**Fig. S6** Linear fitting of Δ*G*° versus *T*.

**Table S1** TC adsorption parameters calculated by pseudo-first-order and pseudo-second-order model.

| Adsorbents | *q*_e,exp_ | pseudo-first-order model | | | | pseudo-second-order model | | |
| --- | --- | --- | --- | --- | --- | --- | --- | --- |
|  |  | *k*_1_ (1/min) | *q*_e,1_ (mg/g) | *R*^2^ | *k*_2_ (g/mg min) | | *q*_e,2_ (mg/g) | *R*^2^ |
| RBC | 40.9 | (1.5±0.2)×10^-2^ | 41.2±0.5 | 0.936 | (5.2±0.5)×10^-4^ | | 42.6±0.3 | 0.984 |
| KMBC | 112.6 | (2.6±0.5)×10^-2^ | 105.8±4.5 | 0.804 | (3.4±0.6)×10^-4^ | | 111.3±3.1 | 0.931 |
| 1% HA/KMBC | 108.3 | (3.6±0.3)×10^-2^ | 99.8±6.6 | 0.873 | (4.1±0.5)×10^-4^ | | 105.6±4.4 | 0.952 |
| 10% HA/KMBC | 72.5 | (5.7±0.9)×10^-3^ | 68.4±2.5 | 0.900 | (1.0±0.1)×10^-4^ | | 76.9±2.2 | 0.967 |

**Table S2** TC adsorption parameters calculated by intra-particle diffusion model.

| Adsorbents | Stage A | | |  | Stage B | | |  | Stage C | | |
| --- | --- | --- | --- | --- | --- | --- | --- | --- | --- | --- | --- |
|  | *k*_d1_  (mg/g min^0.5^) | *C*_a_  (mg/g) | *R*^2^ |  | *k*_d2_  (mg/g min^0.5^) | *C*_b_  (mg/g) | *R*^2^ |  | *k*_d3_  (mg/g min^0.5^) | *C*_c_  (mg/g) | *R*^2^ |
| RBC | 2.07±0.22 | 6.80±1.66 | 0.966 |  | 0.80±0.05 | 19.81±0.89 | 0.990 |  | 0.11±0.03 | 37.12±1.30 | 0.649 |
| KMBC | 4.67±0.49 | 30.76±3.20 | 0.968 |  | 1.65±0.13 | 66.86±2.37 | 0.983 |  | 0.45±0.06 | 96.15±1.98 | 0.919 |
| 1% HAs/KMBC | 4.36±0.31 | 24.85±1.20 | 0.985 |  | 2.40±0.50 | 46.25±10.03 | 0.880 |  | 0.43±0.03 | 92.75±0.92 | 0.981 |
| 10% HAs/KMBC | 2.22±0.76 | 10.11±6.43 | 0.717 |  | 2.44±0.41 | 7.48±7.06 | 0.921 |  | 0.74±0.14 | 44.81±4.64 | 0.848 |

**Table S3** ANOVA results for adsorption amount of TC at different reaction times by different adsorbents.

| Time(min) | RBC | KMBC | 1%HAs/KMBC | 10%HAs/KMBC |
| --- | --- | --- | --- | --- |
| 15 | 13.94±1.02c | 49.46±1.36a | 41.72±0.05b | 14.28±1.59c |
| 30 | 18.49±1.04c | 56.57±1.86a | 48.48±0.39b | 20.01±2.35c |
| 60 | 23.49±0.70d | 63.69±2.00a | 60.51±0.81b | 29.14±0.63c |
| 120 | 28.49±1.11d | 84.16±2.25a | 68.96±1.69b | 32.54±0.89c |
| 240 | 31.58±2.24d | 93.22±2.51a | 83.57±2.64b | 47.57±1.22c |
| 360 | 35.65±1.36d | 100.35±3.47a | 96.51±1.23b | 54.48±0.55c |
| 600 | 39.21±1.05d | 106.73±1.98a | 102.65±1.16b | 59.28±1.98c |
| 840 | 40.23±1.11d | 109.66±2.81a | 105.41±0.89b | 66.01±0.70c |
| 1080 | 41.29±0.55d | 111.48±2.48a | 106.96±1.28b | 69.60±1.12c |
| 1200 | 40.98±1.91d | 112.24±1.36a | 107.59±1.01b | 71.64±0.72c |
| 1440 | 41.35±0.15d | 112.90±2.43a | 109.03±0.75b | 72.57±1.33c |
| 1560 | 40.87±1.50d | 112.64±3.19a | 108.33±0.73b | 72.54±0.83c |

Different letters in the same line indicated statistical significance at p < 0.05.

**Table S4** Adsorption thermodynamic parameters of TC.

| Adsorbents | *T* (K) | ln *K*_0_ | Δ*G*^o^ (kЈ/mol) | Δ*H*^o^ (kЈ/mol) | Δ*S*^o^ (kJ/K mol) |
| --- | --- | --- | --- | --- | --- |
| RBC | 298 K | 0.09±0.02 | -0.22±0.06 | 55.40±2.35 | 0.19±0.01 |
|  | 308 K | 0.81±0.00 | -2.07±0.01 |  |  |
|  | 318 K | 1.63±0.11 | -4.31±0.30 |  |  |
|  |  |  |  |  |  |
| KMBC | 298 K | 1.32±0.01 | -3.27±0.03 | 51.00±2.91 | 0.18±0.01 |
|  | 308 K | 2.18±0.03 | -5.58±0.09 |  |  |
|  | 318 K | 2.62±0.01 | -6.93±0.02 |  |  |
|  |  |  |  |  |  |
| 1% HAs/KMBC | 298 K | 1.27±0.01 | -3.15±0.03 | 51.51±4.28 | 0.18±0.01 |
|  | 308 K | 2.05±0.02 | -5.25±0.06 |  |  |
|  | 318 K | 2.55±0.02 | -6.74±0.06 |  |  |
|  |  |  |  |  |  |
| 10% HAs/KMBC | 298 K | 0.70±0.02 | -1.73±0.04 | 45.61±13.23 | 0.16±0.04 |
|  | 308 K | 1.76±0.01 | -4.51±0.02 |  |  |
|  | 318 K | 2.12±0.00 | -5.60±0.00 |  |  |

**Table S5** ANOVA results for adsorption amount of TC at different temperature by different adsorbents when TC concentration was 50 mg/L.

| Temperature (K) | RBC | KMBC | 1% HAs/KMBC | 10% HAs/KMBC |
| --- | --- | --- | --- | --- |
| 298 | 42.59±0.47d | 112.67±0.01a | 107.38±0.27b | 72.87±0.86c |
| 308 | 77.76±1.15d | 161.21±2.95a | 157.61±1.69b | 132.55±0.07c |
| 318 | 122.80±11.13c | 188.00±1.27a | 188.03±1.63a | 158.17±0.00b |

Different letters in the same line indicated statistical significance at p < 0.05.

| TC concentration | RBC | KMBC | 1% HAs/KMBC | 10% HAs/KMBC |
| --- | --- | --- | --- | --- |
| 50 | 42.59±0.47d | 112.67±0.01a | 107.38±0.27b | 72.87±0.86c |
| 100 | 70.25±1.03d | 152.17±2.84a | 144.57±0.12b | 103.38±4.87c |
| 150 | 94.80±3.34d | 175.90±1.39a | 167.92±4.61b | 129.14±0.53c |
| 200 | 109.18±3.01d | 198.52±6.84a | 188.74±5.72b | 145.06±6.28c |
| 300 | 139.80±5.37d | 238.68±0.90a | 222.30±3.86b | 178.40±8.73c |

**Table S6** ANOVA results for adsorption amount of TC at different initial concentration of TC by four adsorbents at 298 K.

Different letters in the same line indicated statistical significance at p < 0.05.

| pH | RBC | KMBC | 1% HAs/KMBC | 10% HAs/KMBC | KMBC with dissolved HAs |
| --- | --- | --- | --- | --- | --- |
| 5 | 31.64±1.15e | 125.05±0.13b | 118.86±0.41c | 63.57±2.50d | 144.69±2.78a |
| 6 | 40.15±1.61e | 119.51±0.20b | 108.46±2.32c | 70.00±0.21d | 142.14±2.11a |
| 7 | 42.27±1.10e | 118.44±1.46b | 108.48±1.82c | 72.60±2.82d | 132.64±0.76a |
| 8 | 36.87±2.28e | 113.57±0.71b | 95.77±0.22c | 57.10±1.31d | 127.43±0.15a |
| 9 | 33.61±1.48e | 111.40±3.50b | 82.89±2.04c | 45.92±2.39d | 132.15±1.44a |
| 10 | 30.53±0.76d | 102.48±1.28b | 70.65±0.51c | 23.92±5.20e | 123.81±0.75a |

**Table S7** ANOVA results for adsorption amount of TC at different initial pH by different adsorbents.

Different letters in the same line indicated statistical significance at p < 0.05.

**Table S8** ANOVA results for adsorption amount of TC at different concentration of dissolved HAs by RBC and KMBC.

| dissolved HAs concentration (mg/L) | 0 | 0.5 | 1 | 2 | 5 | 10 | 20 |
| --- | --- | --- | --- | --- | --- | --- | --- |
| RBC | 40.87±2.57b | 40.23±1.11b | 40.44±0.48b | 38.89±1.18b | 43.73±1.46a | 23.29±1.43c | 12.28±0.54d |
| KMBC | 112.64±1.12b | 113.91±1.26b | 111.89±2.11b | 114.51±1.01b | 137.94±1.48a | 99.02±1.19c | 72.62±1.63d |

Different letters in the same line indicated statistical significance at p < 0.05.

**Table S9** ANOVA results for TC adsorption by KMBC at different addition sequences of dissolved HAs, TC and KMBC at pH=7.

| addition  sequences  adsorbent | KMBC-(HAs-TC) | (KMBC-HAs)-TC | (KMBC-TC)-HAs |
| --- | --- | --- | --- |
| KMBC | 132.64±0.76c | 116.41±1.67b | 156.09±0.04a |

Different letters in the same line indicated statistical significance at p < 0.05.
